# Supplementary material for: Risk factors for the progression to multimorbidity among UK urban working-age adults. A community cohort study
Source: PLoS One. 2023 Sep 8;18(9):e0291295. doi: 10.1371/journal.pone.0291295 (PMC10490989; doi:10.1371/journal.pone.0291295)
Supplement: Attachment — Submitted filename: Response to reviewers_01.docx [file pone.0291295.s001.docx]

| Institute of Psychiatry, Psychology and Neuroscience |  | Department of Psychological Medicine  Institute of Psychiatry, Psychology and Neuroscience (IoPPN)  16 De Crespigny Park  London SE5 8AF  Telephone 020 7848 0812  Fax 020 7848 4321 |  |  |
| --- | --- | --- | --- | --- |

01 June 2023

PLOS One

Editor-in-Chief

**Title: Risk factors for the progression to multimorbidity among UK urban working-age adults. A community cohort study.**

Dear Editor-in-Chief,

We have now addressed all the points raised by the Editor and Reviewers and have re-submitted the revised manuscript. Of particular note, we have provided additional clarification of the statistical modelling we used as this was not clear. The table below contains details of our responses to feedback from both the editor and the reviewers, and where applicable, details of revisions that have been made to the manuscript.

We have also addressed the additional points raised by the Editor in the email received on 26^th^ May 2023. Our responses are included in the table below.

I look forward to hearing from you in due course.

Your sincerely,

Dr Anne Stagg MSc PhD MBPsS

Research Associate, King’s College London and Guy’s and St Thomas’ NHS Foundation Trust.

Tel 07702 869255 Email: [anne.l.stagg@kcl.ac.uk](mailto:anne.l.stagg@kcl.ac.uk)

| **Long-term health conditions in UK working-age adults: A cross-sectional analysis of associations with demographic, socioeconomic, psychosocial, and health-related factors in an inner-city population** | | |
| --- | --- | --- |
|  | **Editor comments / Journal requirements** | **Response from authors** |
| 1 | Please ensure that your manuscript meets PLOS ONE's style requirements, including those for file naming. The PLOS ONE style templates can be found at [https://journals.plos.org/plosone/s/file?id=wjVg/PLOSOne_formatting_sample_main_body.pdf](https://eur03.safelinks.protection.outlook.com/?url=https%3A%2F%2Fjournals.plos.org%2Fplosone%2Fs%2Ffile%3Fid%3DwjVg%2FPLOSOne_formatting_sample_main_body.pdf&data=05%7C01%7Canne.l.stagg%40kcl.ac.uk%7Cd8c32e3a3ca4458f13e208db3bfdced9%7C8370cf1416f34c16b83c724071654356%7C0%7C0%7C638169733497998506%7CUnknown%7CTWFpbGZsb3d8eyJWIjoiMC4wLjAwMDAiLCJQIjoiV2luMzIiLCJBTiI6Ik1haWwiLCJXVCI6Mn0%3D%7C3000%7C%7C%7C&sdata=xcY0eB5dhUklTKu7WHoIFE4JYB7Ll5D3wCJITzeqozc%3D&reserved=0) and  [https://journals.plos.org/plosone/s/file?id=ba62/PLOSOne_formatting_sample_title_authors_affiliations.pdf](https://eur03.safelinks.protection.outlook.com/?url=https%3A%2F%2Fjournals.plos.org%2Fplosone%2Fs%2Ffile%3Fid%3Dba62%2FPLOSOne_formatting_sample_title_authors_affiliations.pdf&data=05%7C01%7Canne.l.stagg%40kcl.ac.uk%7Cd8c32e3a3ca4458f13e208db3bfdced9%7C8370cf1416f34c16b83c724071654356%7C0%7C0%7C638169733497998506%7CUnknown%7CTWFpbGZsb3d8eyJWIjoiMC4wLjAwMDAiLCJQIjoiV2luMzIiLCJBTiI6Ik1haWwiLCJXVCI6Mn0%3D%7C3000%7C%7C%7C&sdata=Dc2WAECIkClrAsCW8FKKZJy7RlauylXpcjaLN1k7NFY%3D&reserved=0) | We have checked PLOS ONE’s style requirements are applied throughout the document and one adjustment has been made:   1) Table 2 legend has been altered so it is in one section with the footnotes following the legend. 2) There is no longer a supplementary table, so the re-naming of that table is no longer required. 3) Please could you ensure that Prof Ira Madan and Dr Sharon Stevelink are joint last/senior authors. Thank you. |
| 2 | Thank you for stating the following in the Competing Interests section:  "This paper represents independent research part funded by NIHR Maudsley Biomedical Research Centre at South London and Maudsley NHS Foundation Trust and King’s College London, and the NIHR. The views expressed are those of the authors and not necessarily those of the NHS, the NIHR or the Department of Health and Social Care."  Please confirm that this does not alter your adherence to all PLOS ONE policies on sharing data and materials, by including the following statement: "This does not alter our adherence to  PLOS ONE policies on sharing data and materials.” (as detailed online in our guide for authors [http://journals.plos.org/plosone/s/competing-interests](https://eur03.safelinks.protection.outlook.com/?url=http%3A%2F%2Fjournals.plos.org%2Fplosone%2Fs%2Fcompeting-interests&data=05%7C01%7Canne.l.stagg%40kcl.ac.uk%7Cd8c32e3a3ca4458f13e208db3bfdced9%7C8370cf1416f34c16b83c724071654356%7C0%7C0%7C638169733497998506%7CUnknown%7CTWFpbGZsb3d8eyJWIjoiMC4wLjAwMDAiLCJQIjoiV2luMzIiLCJBTiI6Ik1haWwiLCJXVCI6Mn0%3D%7C3000%7C%7C%7C&sdata=Z5%2BLSTgLdwVmI2DaVKprelBcKannWJ%2Bt2lv4O6%2BbkjU%3D&reserved=0)).  If there are restrictions on sharing of data and/or materials, please state these. Please note that we cannot proceed with consideration of your article until this information has been declared.  Please include your updated Competing Interests statement in your cover letter; we will change the online submission form on your behalf. | Please could you update our Competing Interests statement with the following statement which includes the reference to PLOS ONE policies on sharing data and materials:  “This paper represents independent research part funded by NIHR Maudsley Biomedical Research Centre at South London and Maudsley NHS Foundation Trust and King’s College London, and the NIHR. The views expressed are those of the authors and not necessarily those of the NHS, the NIHR or the Department of Health and Social Care. This does not alter our adherence to PLOS ONE policies on sharing data and materials.” |
| 3 | We noted in your submission details that a portion of your manuscript may have been presented or published elsewhere. "The supporting information table listing the prevalence of health conditions at both timepoints has been published in a preliminary study which has recently been published in BMJ Open.  However, it is also key information for this paper in relation to the number of individual long-term conditions at baseline and at follow-up as it was used to create the long-term condition variables showing acquisition of a first LTC between the two timepoints, and also development of multimorbidity over the same timeframe. Since it has been published before, it was included only as a supplementary table so readers could easily refer to it." Please clarify whether this [conference proceeding or publication] was peer-reviewed and formally published. If this work was previously peer-reviewed and published, in the cover letter please provide the reason that this work does not constitute dual publication and should be included in the current manuscript. | We can confirm that the supplementary Table S1 has been published in the following peer-reviewed preliminary study:  Stagg AL, Hatch S, Fear NT, et al. Long-term Health conditions in UK working-age adults: a cross-sectional analysis of associations with demographic, socioeconomic, psychosocial and health-related factors in an inner-city population. BMJ Open 2022;12:e062115. doi:10.1136/bmjopen-2022-062115  The reason this table was included as a supplementary table was as previously stated, for ease of access. However, this has now been amended so that reference is now made to the initial publication where readers can access the full information about the numbers of conditions at both timepoints (see Revised Manuscript with Track Changes, page 7, lines 140 and 141). The original supplementary file is therefore no longer necessary. The supporting information section on page 27, lines 506-517 has also been removed. |
| 4 | In your Data Availability statement, you have not specified where the minimal data set underlying the results described in your manuscript can be found. PLOS defines a study's minimal data set as the underlying data used to reach the conclusions drawn in the manuscript and any additional data required to replicate the reported study findings in their entirety. All PLOS journals require that the minimal data set be made fully available. For more information about our data policy, please see [http://journals.plos.org/plosone/s/data-availability](https://eur03.safelinks.protection.outlook.com/?url=http%3A%2F%2Fjournals.plos.org%2Fplosone%2Fs%2Fdata-availability&data=05%7C01%7Canne.l.stagg%40kcl.ac.uk%7Cd8c32e3a3ca4458f13e208db3bfdced9%7C8370cf1416f34c16b83c724071654356%7C0%7C0%7C638169733497998506%7CUnknown%7CTWFpbGZsb3d8eyJWIjoiMC4wLjAwMDAiLCJQIjoiV2luMzIiLCJBTiI6Ik1haWwiLCJXVCI6Mn0%3D%7C3000%7C%7C%7C&sdata=r1hQ%2FwpW2Z7FmcMZYL3mo25FO%2FqjO%2FYcth1dja992ks%3D&reserved=0).  Upon re-submitting your revised manuscript, please upload your study’s minimal underlying data set as either Supporting Information files or to a stable, public repository and include the relevant URLs, DOIs, or accession numbers within your revised cover letter. For a list of acceptable repositories, please see [http://journals.plos.org/plosone/s/data-availability#loc-recommended-repositories](https://eur03.safelinks.protection.outlook.com/?url=http%3A%2F%2Fjournals.plos.org%2Fplosone%2Fs%2Fdata-availability%23loc-recommended-repositories&data=05%7C01%7Canne.l.stagg%40kcl.ac.uk%7Cd8c32e3a3ca4458f13e208db3bfdced9%7C8370cf1416f34c16b83c724071654356%7C0%7C0%7C638169733497998506%7CUnknown%7CTWFpbGZsb3d8eyJWIjoiMC4wLjAwMDAiLCJQIjoiV2luMzIiLCJBTiI6Ik1haWwiLCJXVCI6Mn0%3D%7C3000%7C%7C%7C&sdata=itT47IHZYgqA3zRIwtx0ezsDAMTbvrD%2F3JC8ctZKBGs%3D&reserved=0). Any potentially identifying patient information must be fully anonymized.  Important: If there are ethical or legal restrictions to sharing your data publicly, please explain these restrictions in detail. Please see our guidelines for more information on what we consider unacceptable restrictions to publicly sharing data: [http://journals.plos.org/plosone/s/data-availability#loc-unacceptable-data-access-restrictions](https://eur03.safelinks.protection.outlook.com/?url=http%3A%2F%2Fjournals.plos.org%2Fplosone%2Fs%2Fdata-availability%23loc-unacceptable-data-access-restrictions&data=05%7C01%7Canne.l.stagg%40kcl.ac.uk%7Cd8c32e3a3ca4458f13e208db3bfdced9%7C8370cf1416f34c16b83c724071654356%7C0%7C0%7C638169733497998506%7CUnknown%7CTWFpbGZsb3d8eyJWIjoiMC4wLjAwMDAiLCJQIjoiV2luMzIiLCJBTiI6Ik1haWwiLCJXVCI6Mn0%3D%7C3000%7C%7C%7C&sdata=zbdrMjdZS78nrrKjjUUwBCE2dTBnZRrv7vu7kAK4JL8%3D&reserved=0). Note that it is not acceptable for the authors to be the sole named individuals responsible for ensuring data access.  We will update your Data Availability statement to reflect the information you provide in your cover letter. | Data Availability:  This study used third party data from the SELCoH survey. The following data availability statement should be included:  SELCoH data is available to researchers upon request. Please contact [selcoh@kcl.ac.uk](mailto:selcoh@kcl.ac.uk) to apply for access. For more information visit [www.kcl.ac.uk/research/selcoh](https://eur03.safelinks.protection.outlook.com/?url=http%3A%2F%2Fwww.kcl.ac.uk%2Fresearch%2Fselcoh&data=05%7C01%7Canne.stagg%40kcl.ac.uk%7C62a0d0605e3c42ff7a7608db41070e38%7C8370cf1416f34c16b83c724071654356%7C0%7C0%7C638175270786268070%7CUnknown%7CTWFpbGZsb3d8eyJWIjoiMC4wLjAwMDAiLCJQIjoiV2luMzIiLCJBTiI6Ik1haWwiLCJXVCI6Mn0%3D%7C3000%7C%7C%7C&sdata=1YaX9%2BEMLXk70J4%2FXXjJRvVHAMKNiGf36VVVn3aQi3w%3D&reserved=0) |
|  | **Further** **Editor comments / Journal requirements (Email: 26 05 23)** |  |
| 1 & 2 | We've checked your submission and before we can proceed, we need you to address the following issues:  1. We note that your uploaded Cover Letter is not a Cover Letter but a Response to Reviewers file. It is important that you include a cover letter with your manuscript. Please ensure that this letter is addressed specifically to PLoS ONE. Please also include  * why this manuscript is suitable for publication in PLoS ONE.  * how does your paper provide a worthwhile addition to the scientific literature?  * how does your paper relate to previously published work?  * which types of scientists do you believe will be most interested in your study?  Please also note that the Cover Letter and Response to Reviewers file should be uploaded as two separate files.  2. Please upload a Response to Reviewers letter which should include a point by point response to each of the points made by the Editor and / or Reviewers. (This should be uploaded as a 'Response to Reviewers' file type.) Please follow this link for more information: <https://eur03.safelinks.protection.outlook.com/?url=http%3A%2F%2Fblogs.plos.org%2Feveryone%2F2011%2F05%2F10%2Fhow-to-submit-your-revised-manuscript%2F&data=05%7C01%7Canne.l.stagg%40kcl.ac.uk%7C0cb97bc2fa5d4fcb1fb908db5d96cff3%7C8370cf1416f34c16b83c724071654356%7C0%7C0%7C638206674534014930%7CUnknown%7CTWFpbGZsb3d8eyJWIjoiMC4wLjAwMDAiLCJQIjoiV2luMzIiLCJBTiI6Ik1haWwiLCJXVCI6Mn0%3D%7C3000%7C%7C%7C&sdata=upX54CLXyfauygwUSl3vmgiwyj368IvaEQrw5KAAuCY%3D&reserved=0> | The cover letter and Response to Reviewers letters have now been uploaded as two separate files. |
| 3 | Your ethics statement should only appear in the Methods section of your manuscript. If your ethics statement is written in any section besides the Methods, please move it to the Methods section and delete it from any other section. Please ensure that your ethics statement is included in your manuscript, as the ethics statement entered into the online submission form will not be published alongside your manuscript. | The ethics statement has now been moved within the Methods from the Statistical analyses section to the Study design, setting and participants section (see Revised Manuscript with Track Changes page 6 lines 125-128). Thank you for pointing this out as this is a more appropriate section for the ethics statement which is linked to the SELCoH data collection. |
| 4 | We note your current Data Availability Statement: "This study used third party data from the SELCoH survey. The following data availability statement should be included:  SELCoH data is available to researchers upon request. Please contact [selcoh@kcl.ac.uk](mailto:selcoh@kcl.ac.uk) to apply for access. For more information visit <https://www.kcl.ac.uk/research/selcoh>  Regarding third party data, please confirm whether or not the authors received any special privileges in accessing the data that other researchers would not have. | We can confirm that the authors did not receive any special privileges in accessing the data that other researchers would not have. |
|  | **Reviewer 1 Comments to the Author** |  |
|  | This is an interesting and well-written paper. Its strengths include the longitudinal data and the data on employment - which is particularly valuable in the context of multimorbidity among working-age people. Specific comments below | We thank the first reviewer for their comments on the paper and have responded to their specific points below. |
| 1 | The model description is not 100% clear. It would be good to know the parameterisation and any assumptions. I also wonder if it would be easier to run two separate logistic regression models. One for any LTC and then another, conditional on having a LTC what is the odds of developing further LTCs. This would make it difficult to model improvement, but the trade off is it would be simpler | We agree with the reviewer model description should be made clearer, and a similar comment on clarity was also raised by the second reviewer. To address this, the model description in the transcript has been revised in the statistical analyses section to make it clearer (see document ‘Revised Manuscript with Track Changes’, page 10 lines 209-217) and page 14 lines 253-254. In explanation to the reviewers, we used multinomial logistic regressions and with the dependent outcome variable consisting of the following categories: (1) the odds of developing any number of LTCs at the second timepoint having not had any LTCs at baseline, and (2) when there is one LTC at baseline, the odds of developing further LTCs by the second timepoint. (3) No reported LTCs at either S1 or S2 was used as the reference category in the regressions. The outcome is therefore similar to the two models the reviewer suggests but has the advantage of including the 479 individuals in the healthy group who had no LTCs at either timepoint (the reference group), is that it increases the strength of the analysis compared to running two separate regression models where we would lose data from these 479 individuals. Since we note that the second reviewer was satisfied with the statistics used, we have not changed our modelling, but are happy to reconsider if the editor believes that re-modelling the analysis is essential. |
| 2 | There seem to be a lot of parameters for the relatively small number of events (I counted 39 even though there were only 46 people who transitioned from 1 to many LTCs). I know that the rule of thumb 10 events per variable is an overstatement, but 39 still seems excessive. Moreover, age could be modelled using splines or fractional polynomials to reduce the number of parameters that need to be fitted. | This comment regarding the balance between the number of parameters for the number of participants appears to be linked to the previous comment about lack of clarity over the model description as it would be highly relevant where all the predictor variables were tested simultaneously within in the same model. However, we did not carry out a multiple regression, and each predictor variable was tested independently for the multinomial LTC outcomes, apart from the adjusted model which included age and gender, so a maximum of 3 predictor variables in the adjusted model. |
| 3 | Related to this, if I understand correctly these coefficients are all mutually adjusted (even for the unadjusted model). This leads to the table 2 fallacy (<https://pubmed.ncbi.nlm.nih.gov/23371353/>). Instead it would be better to draw some DAGS and decide on which covariates should go into models asking questions which are relevant for clinical practice or for determining policy. | This comment has been addressed in the response to this reviewer’s the first comment as it relates to the model description, which we have now clarified and explained that the coefficients are not all mutually adjusted. |
| 4 | Notwithstanding the model fitting some post-processing to obtain absolute risks of transitioning to each state for people with different covariate values would be useful. The odds ratio scale is fine for relative effects, but less good for describing patterns of disease and risks, which is the purpose here. | Again, we think that this comment is linked to the lack of clarity about the regression modelling we used. We hope that this has now been addressed with the revisions that we have made. |
| 5 | It would be useful to discuss why people were not followed-up in the second round. Could this be due to ill health preventing them from completing the wave? It would be good to report the number and characteristics of people who were invited for a second wave but did not participate. | The second reviewer has also raised a similar comment about reasons for lack of response at follow-up particularly in relation to ill health, which we have addressed below in response to their comment. |
| 6 | In the discussion the statement is made that the odds ratios are different for 0/1 versus 1/many LTCs. If this is to be asserted the difference in the odds ratios should be estimated. Again, however, some thought needs to be given to scale. Some characteristics may increase risk MORE or LESS on the absolute scale while being the same on the relative scale (and vice versa). | We were a little uncertain as to the interpretation of this comment, but assume that it is in reference to the following sentence (page 19, lines 333-336): *Whilst the risk of progression from good health to multimorbidity increased with age, this study showed that even in the younger age group (35 to 44 years), there was a greater risk of progressing from one to multiple LTCs, even when compared to developing a first LTC.*  This sentence primarily relates to the greater risk of progressing from one to multiple LTCs at the younger age (35-44 years) group which was shown to be statistically significant, whilst the risk of the initial development from none to one-or-more LTCs in the same age group was not statistically significant in both the unadjusted and adjusted (by gender) models. In addition, the relative risk ratios increase as age increases and this indicates an increased risk of developing LTCs with age. It may be that the reviewer was again considering the model to be a multiple logistic regression, which has now been clarified to not be the case.  We have added in a comment on page 19 line 334-335 that this increased risk is statistically significant. However, if this is not the correct interpretation of the reviewer’s comment, please could you provide more clarification so that we can make the appropriate revision. |
| 7 | Would be good to see some numbers in the abstract results section. | The authors would have like to have a included some numbers in the abstract section, but the word count limitation prevented this. |
| 8 | The introduction states that having a single LTC occurs before multiple LTCs - isn't this almost true by definition (at least for sufficiently granular and complete data)? | The authors agree with the comment that the development of multiple LTCs from 1 LTC is a common fact. The reason it is included in the introduction is because the paper is looking at risk factors for progressing from one to MLTCs, so this comment was made as the initial part of the argument justifying this study which identifies risk factors linked to this progression. |
| 9 | nice to see the number of events in table 1. Would be good to see this at the top of Table 2 also. | If the numbers for Table 2 were put at the top of the table, it would not show the reference group of 479 participants, so we have inserted the numbers against each of the indicator variables (pages 14-16. This also then indicates where the small number of missing records lie (see also the comment about missing data on page 9, line 201-202 in the document ‘Revised Manuscript with Track Changes’). |
| 10 | Not sure of the usefulness of the unadjusted coefficients in table 2 | This comment is indeed understandable if all the coefficients had been tested together, as they would not be unadjusted results. However, having revised the script to provide a clearer explanation of the modelling used, we hope that now the inclusion of the unadjusted values in this table makes sense since all the predictor variables were tested individually. The adjusted value columns then show results where each predictor variable was adjusted by age and gender so that a direct comparison can be made between the unadjusted and adjusted relative risk ratios. |
| 11 | Would be good to share code and (if possible) aggregate level data (I know IPD cannot be shared). | As acknowledged by the reviewer, individual participant data cannot be directly shared. However, access to the SELCoH data is available and details have now been provided (see the updated data availability statement).  Whilst the entire coding process covers data cleaning and another preliminary study, so not all relevant for this paper, useful examples of the code used for the unadjusted and adjusted regressions (using marital status as an example) are:  svy:mlogit ltc_change_3gps i.relationship_s1,rrr  svy:mlogit ltc_change_3gps i.relationship_s1 i.sex age_s1cont ,rrr  This code should also help to clarify for the reviewers the modelling we used for the regressions. |
|  | **Reviewer 2 comments** |  |
|  | The manuscript “Risk factors for the progression to multimorbidity among UK urban working-age adults. A community cohort study” explores factors associated with the initiation and progression of long-term chronic conditions among the working population in the UK. This is a very nice paper with an important contribution to the literature. The manuscript is well written and organized. The methodology and analytical methods used are appropriate for the study goals and results are presented with clarity. I only have minor questions/clarifications as follows. | We thank the reviewer for their kind comments regarding the manuscript and have addressed their specific points below. |
| 1 | Page 6, line 116-124: The analytical sample included participants who completed both surveys. This is understandable given the objectives. However, I wonder if not responding at wave 2 may partially related to chronic conditions or some important participant characteristics. Could the authors comment on the differences in # of conditions and characteristics of those excluded due to loss to follow up? I think a brief comment in the discussion/limitations should suffice. | This is an interesting comment, and whilst the cleaned data set used in this study did not include this group, the authors have investigated the SELCoH data for health-related information about those who did not respond at follow-up. 21 participants were ineligible for the second wave due to death or poor health. However, the data used for the current study was restricted to participants under the age of 64 at follow-up as it focused on working-age participants, and none of these 21 participants were in this age group. Consequently, we would be unable to comment further on this as an actual limitation within our study. |
| 2 | Page 10, lines 204-210: It is not clear how adjusted models were fit. For example, for BMI, the adjusted model included BMI, age, sex only or the RRR is from a model with all variables? Please clarify. If the latter, please comment on the possible impact of collinearity to the results. | The comment by this reviewer highlighting the need for clarity regarding the modelling has been addressed in the response to the first reviewer who also raised this (please see the response to reviewer 1, comment 1). The description in the methods section has now been altered (see Revised Manuscript with Track Changes page 10 lines 209-217). In summary, the multinomial LTC trajectory variable was regressed on each indicator variable individually. Two models were tested 1) these regressions were carried out unadjusted and 2) then adjusted by gender and age. |
