# Supplementary material for: Risk factors for the progression to multimorbidity among UK urban working-age adults. A community cohort study
Source: PLoS One. 2023 Sep 8;18(9):e0291295. doi: 10.1371/journal.pone.0291295 (PMC10490989; doi:10.1371/journal.pone.0291295)
Supplement: Attachment — Submitted filename: Response to Editors.docx [file pone.0291295.s002.docx]

**Response to the additional comments from the editors**

**Note:** All references to the location of revisions made to the manuscript relate to the tracked changes version.

1. **Editor comment:**

General: Please accommodate the request from Reviewer One to provide estimates in the abstract. Currently, the abstract contains excess information that could be condensed to make space for these required amendments.

**Response from authors:**

The abstract has been condensed and relative risk ratios (RRRs) with 95% confidence intervals have been provided for all the significant unique risk factors for both initiation and progression pathways (Abstract pages 3 and 4, lines 49-73). The abstract is still within the maximum 300 word count requirements.

2. **Editor comment:**

We also ask that you revise your manuscript to refrain from using causal language (e.g., "determine"), as the present study design does not permit assertions of causality.

**Response from authors:**

The use of ‘determinants’ is related to the longitudinal design of the study which has two timepoints with the baseline variables being tested against two trajectories of risk of developing LTCs (This was acknowledged by Reviewer 1 in their comments to the authors who cited the longitudinal data as a strength of the study). However, it is acknowledged that there is an over-use of ‘determinants’ in the paper and a lack of caution in interpreting the results, so the following revisions have been made:

Revisions so that there is the appropriate use of the term ‘variables’ rather than ‘determinants’:

- Abstract, page 3 line 53: ‘*determinants’* changed to ‘*variables’*
- Methods, page 7 line 149: The heading has been revised from ‘*Determinant* *variables’* to ‘*Independent* *variables’*.
- Methods page 8 line 175: ‘*determinants’* changed to ‘*variables’*
- Table 1 pages 11-14: ‘*determinants’* changed to ‘*variables’* and Table 2 (pages 14-17) has been changed to match the terminology used in Table 1.
- Discussion: Page 22 line 397: ‘*determinant* *variables’* has been amended to ‘*independent* *variables’*
- Discussion: Page 22, line 399 ‘*determinants’* has been changed to ‘*variables’*.

Additional changes:

- Abstract page 4 line 70: ‘d*eterminants’* has been changed to '*risk factors’*.
- Methods page 14 line 256: ‘*Determinants’* has been changed to ‘*Risk* *factors’*
- Discussion page 19 lines 321-322: The following sentence has been changed from *‘This study indicates that the journey to multimorbidity has both common and unique determinants ..’* to *‘This study provides evidence that the journey to multimorbidity may have both common and unique risk factors..’*
- Discussion page 22 lines 385-387: The following sentence has been changed from ‘*Whilst there may be different determinants of LTC initiation and then progression to multimorbidity, it is of interest that there were common determinants of small social support networks …’* to ‘*It is of interest that whilst there may be different risk factors linked to LTC initiation and then progression to multimorbidity, there were common factors such as small social support networks*...’
- Discussion page 22 lines 402-403: The following sentence has been changed from ‘*If negative health perceptions at S1 are determinants of developing initial LTCs at S2*,..’ to ‘*If negative health perceptions at S1 are associated with the development of initial LTCs at S2*,..’
- Discussion page 23 lines 418-419: ‘*determinants of’* has been changed to ‘*factors supporting’.*
- Discussion page 23 line 428: ‘*determinants of*’ has been changed to ‘*factors linked to*’.

Revisions highlighting caution in interpreting the results:

- The insertion of the following two sentences in the discussion, page 23, lines 421-426: ’*It should also be acknowledged that whilst this study is longitudinal in design with two timepoints, the testing of baseline determinants of the two negative LTC trajectories should be interpreted with caution, and can only imply causality rather than prove it*. *Also, reverse causality cannot be excluded, particularly in the case of employment where health status may lead to reduced working hours or unemployment.’*
- Discussion page 23 lines 427-428: this sentence has been amended so the conclusions are not stated so strongly. It has been changed from: ‘*In conclusion, this study demonstrates that the journey to multimorbidity is complex, with both common and unique determinants of the two stages of* …’ to ‘*In conclusion, this study demonstrates that the journey to multimorbidity is complex, and suggests there are both common and unique factors linked to the two stages of …’.*
- Discussion page 24, lines 432-33: ‘*Whilst we have shown that not being employed full-time can have a negative influence on health*, ..’ has been changed to ‘*Whilst we have shown that not being employed full-time may have a negative influence on health*,..’.

**INTRODUCTION**

3. **Editor comment:**

We advise that you abbreviate the introduction to keep its focus consistent with the aim of the study. Given that the study's objective is exploratory, it appears excessive space is allocated to specific factors, such as occupation. Please shorten the introduction accordingly and realign it with the study's aims.

**Response from authors:**

This manuscript is the outcome of a project supported by a research grant that was very specific in its objectives - to explore risk factors for developing LTCs and progression to multimorbidity in a working-age population. Employment status was specifically included within a wide range of exploratory variables with the additional objective for employment status to receive specific focus as a secondary aim. The current introduction reflects these objectives, and we cannot see how any further amendments would benefit this section. We would like to highlight the comment made by the first reviewer in relation to the usefulness of including employment data (“*This is an interesting and well-written paper. Its strengths include the longitudinal data and the data on employment - which is particularly valuable in the context of multimorbidity among working-age people”*).

**METHODS**

4. **Editor comment:**

It is necessary to provide a clearer account of those individuals who did not participate in the second wave of the study and were subsequently excluded. We recommend including a flowchart as a supplementary file, outlining the participant trajectory. Moreover, we ask that you supply a table comparing the baseline characteristics of participants who met the inclusion criteria but did not participate in the follow-ups.

**Response from authors:**

The data set used for this secondary data analysis study only included participants who had completed both the SELCoH 1 and SELCoH 2 surveys and were within a working-age range. Analysis of the SELCoH data not included in our dataset was outside the aims and objectives of this study. However, there have been many previous SELCoH studies published with information on the original data, and the participant trajectory between SELCoH 1 and SELCoH 2 has been previously reported in studies using the complete SELCoH dataset (For example, Hatch et al., 2016; doi:10.1007/s00127-016-1191-x, and Harber-Aschan et al., 2019; doi: [10.1016/j.jpsychores.2018.11.005](https://doi.org/10.1016/j.jpsychores.2018.11.005)). Hatch et al., (2016) also has a supplementary table comparing baseline demographic characteristics of the SELCoH 1 and SELCoH 2 participants. We have now inserted the following sentence into the methods so that these papers are now referred to (the references have also been adjusted accordingly):

Methods page 6, lines 125-127:

*Demographic characteristics of participants who responded at both waves, and reasons for non-response at the second wave have previously been reported [14, 15].*

Please also see our previous response to the second reviewer in regard to participants who did not complete SELCoH 2, as we were able to establish that the 21 non-responders for health reasons or death (referred to in Harber-Aschan et al., 2019) were outside the age range of our study so would not have been included in any analysis of non-responders

5. **Editor comment:**

We noted that you group certain conditions together that are treated as a single condition in your analysis, as per Stagg et al. (2022). In this regard, please provide a rationale for the validity of classifying subjects as having a single condition when they may have multiple LTCs. Addressing this issue is crucial, as it may necessitate significant alterations in your analysis.

**Response from authors:**

This is a very good point, and we agree that this needs further clarification as no subject was classified as having one condition when they had multiple conditions, and this is not clearly stated in the methods section. During the lengthy LTC data coding process, careful attention was given to the number of individual self-reported conditions when developing the final groupings of conditions to ensure that an individual with two or more conditions always had these conditions listed separately, and not within a single condition group. Once the list of LTC groups were established, a further check was made within Stata by viewing all the condition groups against each participant ID to confirm that the number of conditions within each group for each participant was never more than 1. This process was essential to determining the correct number of LTCs self-reported by each participant.

To ensure clarity on this point, we have amended the long-term conditions section of the methods as follows:

Methods page 7, lines 140-144**.** This sentence has been amended so it now reads: ‘*The final list of LTCs was constructed by allocating conditions either into specific condition groups (for example, two condition groups were asthma and high blood pressure), or into groups of similar conditions where individual case numbers were <5 (for example, gastrointestinal conditions and respiratory conditions).’*

Methods page 7, line 144-145: The following sentence was also inserted: ‘*No participant had more than one condition within any of the individual combined condition groups*.’

6. **Editor comment:**

Your exclusion of individuals whose health status improves may introduce significant selection bias. The current emphasis on studying individuals with a deteriorating health status does not sufficiently justify your choice of analysis. We strongly suggest revising your analysis to include all participants.

**Response from authors:**

Whilst ‘improvers’ were identified in the data and was discussed in the research team as it is interesting, they were not included as an additional LTC trajectory group primarily as this group was never included in the study protocol, and we were confident that excluding it was not detrimental to the key aims of the study focusing on participants reporting increased numbers of LTCs (this was acknowledged in the methods section, page 9, lines 207-209). The ‘improvers ‘group was also complex and comprised sub-groups with some participants reporting 1 LTC at S1 and none at S2, and another group reporting more than 1 LTC at S1 and fewer LTCs at S2. There were additional potential limitations in analysing this group as we did not know whether they improved due to successful on-going treatments, or had experienced a complete recovery, or were in remission (this included cancer) which would have been a major limitation to the findings.

From a statistical perspective, the multinomial regression used in the study comprises the two discrete categorical outcome groups (0 to ≥1 LTCs and 1-to-many LTCs), and inclusion of the improvers group would not have impacted the results of these two categorical outcomes, or imply an error in the results of these two categories due to selection bias.

However, had this study focused more generally on all LTC trajectories (and included comparative patient medical records), a significantly larger sample size would have enabled an in-depth stratified analysis of the ‘improved’ groups of participants. We have acknowledged the omission of this group in the limitation section of the discussion, and have added in ‘*and the addition of medical record data*’:

Discussion page 23, lines 417-421: “ *A larger sample size and the addition of medical record data would also enable factors supporting positive health transitions to be investigated.* *By expanding the scope of future studies to include positive health transitions, this would provide guidance for health promotion interventions aimed at slowing the journey to multimorbidity*”.

**DISCUSSION**

7. **Editor comment:**

Please refine the structure of your discussion for improved readability. For instance, the correlation between employment categories is discussed at two different points (lines 318 and 338). Consolidating this discussion into a single paragraph would greatly enhance readability.

**Response from authors:**

The discussion has now been extensively revised, with the employment section consolidated into a single paragraph and various additional edits made to enhance readability through this section. Please see the tracked changes in the **discussion pages 19-23.**

8. **Editor comment:**

Also, we recommend limiting speculative conclusions, as your study merely observes associations (e.g., employment can both be a potential causal factor for multimorbidity, or employment could reflect health status at baseline which is the real risk factor for multimorbidity).

**Response from authors:**

The revisions to the discussion have taken this point into consideration and made appropriate changes so that speculative conclusions are limited. Please see the changes made to the discussion listed in our response to editor comment 2, as these two comments are linked.

The revisions to the discussion also specifically ensure that there is no suggestion of causality in relation to the regression results between employment and the LTC trajectories. We are aware that there may be a bi-directional association between employment and health and acknowledged this in the following sentence in the discussion:

Discussion page 21, lines 358-360: ‘*One possibility is that there is a higher proportion of people with one LTC at baseline who found the need to change from full-time to part-time employment, and so this group may already be at increased risk*.’

This has been further highlighted in the limitations section of the discussion with the insertion of the following sentence:

Discussion page 23 lines 424-426: ‘*Also, reverse causality cannot be excluded, particularly in the case of employment where health status may lead to reduced working hours or unemployment’.*

9. **Editor comment:**

Also, please reconsider carefully your limitation statement. Consider for example the limited number of LTCs, the grouping of LTCs, the lack of information on propensity to seek care (and thus receive a diagnosis).

**Response from authors:**

The limitations section has been expanded and the following comment inserted into the discussion on page 23, lines 411-417.

*‘In contrast to many studies using patient records, all self-reported conditions were included in this study. However, a larger sample size would have enabled a larger number of LTCs to be analysed separately rather than being combined into similar condition groups. Additionally, despite the advantages of including all self-reported conditions, it should also be noted that without access to medical records, we were unable to confirm whether a formal diagnosis had been received for all conditions, or if there were un-reported conditions that had not yet received a diagnosis or were being successfully managed by the individual.*’
